# Tetraploid Induction by Colchicine Treatment and Crossing with a Diploid Reveals Less-Seeded Fruit Production in Pointed Gourd (*Trichosanthes dioica* Roxb.)

**DOI:** 10.3390/plants9030370

**Published:** 2020-03-17

**Authors:** Jahidul Hassan, Ikuo Miyajima, Yukio Ozaki, Yuki Mizunoe, Kaori Sakai, Wasimullah Zaland

**Affiliations:** 1 Graduate School of Bioresource and Bioenvironmental Sciences, Faculty of Agriculture, Kyushu University, Fukuoka 819-0395, Japan; wasims2000@gmail.com; 2 Institute of Tropical Agriculture, Kyushu University, Fukuoka 819-0395, Japan; imiyajima@agr.kyushu-u.ac.jp; 3 Laboratory of Horticultural Science, Kyushu University, Fukuoka 819-0395, Japan; y.ozaki.255@m.kyushu-u.ac.jp (Y.O.); mizunoe@agr.kyushu-u.ac.jp (Y.M.); 4 Laboratory of Agricultural Ecology, Kyushu University, Fukuoka 819-0395, Japan; sakai@farm.kyushu-u.ac.jp

**Keywords:** colchicine, cucurbits, dioecious, flow cytometry, polyploidization, seed, seedless, tetraploid, *Trichosanthes dioica*

## Abstract

Pointed gourd (*Trichosanthes dioica* Roxb.) (2n = 2x = 22) is a dioecious cucurbit vegetable and green fruit that is edible after cooking. Consumers prefer to consume seedless or less-seeded fruit because seeds are unpalatable due to their hard coats. Therefore, the cross compatibility between the diploid and induced tetraploid will be helpful for seedless or less-seeded fruit production. Thus, the present study was conducted using mature seeds that were immersed in 0.05%, 0.1%, and 0.5% colchicine for 24, 48, and 72 h to induce tetraploids. These tetraploids were used as parents (male or female) in the inter-ploidy and intra-ploidy crosses. A flow cytometric analysis confirmed the induction of three tetraploids at 0.5% colchicine for 48 and 72 h soaking periods. Among these, two (2) females and one (1) male were differentiated after flower initiation. Crossing between the tetraploid’s maternal and diploid paternal parent (4x × 2x), which were revealed to be compatible, resulted in a similar fruit set rate and shape as those of the diploid. In addition, a seed number of 4x × 2x produced fruits that were drastically reduced to 1.8 seeds per fruit, whereas the natural diploid fruits had 26.4 seeds per fruit. These findings suggest that colchicine-induced tetraploid females are important genetic resources for less-seeded fruit production. The genetic stability of tetraploid clones can easily and effectively be maintained by vine cutting for advanced uses.

## 1. Introduction

**Figure 1:**
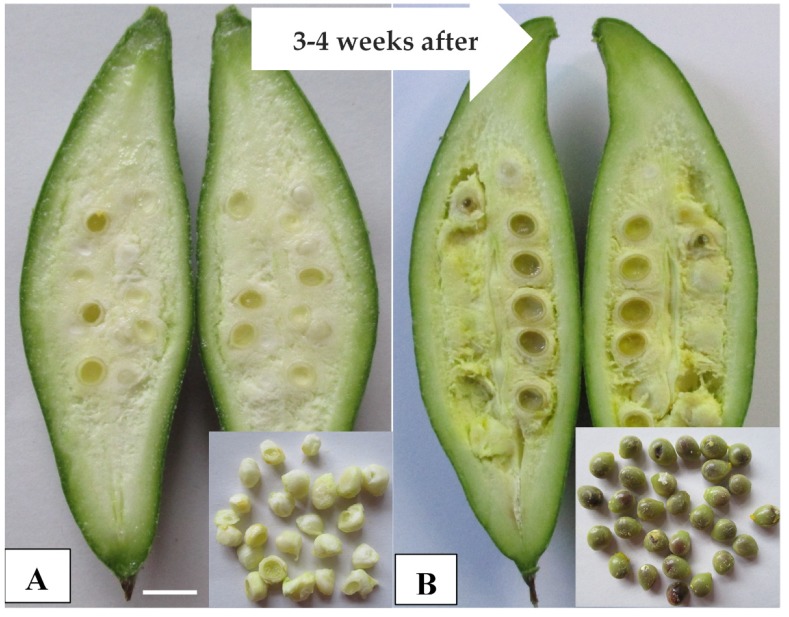
Fruits at the edible stage with soft-coated seeds (**A**) and hard-coated seeds (**B**). The bar indicates 1 cm.

Pointed gourd (*Trichosanthes dioica* Roxb.) is one of the most important cucurbit summer vegetables in India and Bangladesh. Genetically, it is a diploid type with a chromosome number of 2n = 2x = 22, where 11 bivalents are observed in the pollen’s mother cells [1]. Botanically, it is a dioecious type, where the male and female flowers are carried on separate plants. Due to its dioecism, cross pollination is inevitable for fruit setting. Immature green fruits containing seeds with soft coats are edible after cooking. Usually, each fruit contains more than 20 seeds and becomes harder at 3–4 weeks after pollination (Figure 1). At this point, it loses consumer acceptance because of its unpalatable seeds with hard coats. Parthenocarpy induction approaches might be able to solve this problem. 

**Figure 2:**
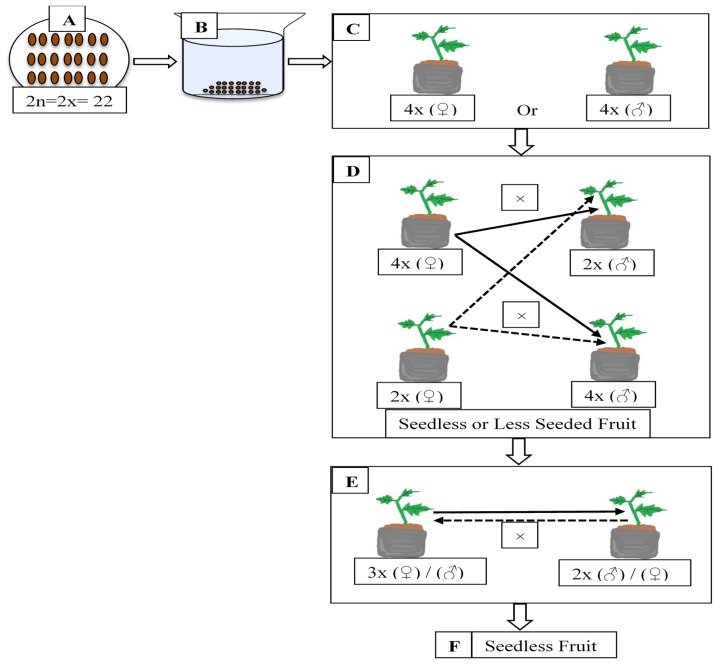
Hypothetical pathways of polyploidization and triploid breeding for seedless fruit production in pointed gourd (*Trichosanthes dioica* Roxb.). (**A**) Mature seeds of diploids (2n = 2x = 22), (**B**) colchicine treatment on seeds, and (**C**) tetraploid induction for either female or male as dioecious and represented by 4x (♀) and 4x (♂), respectively; (**D**) tetraploid female 4x (♀) pollinated with diploid 2x (♂) and tetraploid 4x (♂) males, indicated by straight lines; and diploid females 2x (♀) pollinated with tetraploid 4x (♂) and diploid 2x (♂) males, indicated by dash lines. (**E**) If viable seeds are produced by crossing with the tetraploid parents (female or male), then the F1 progenies will be triploid (3x). The generated triploid female 3x (♀) will be pollinated by a diploid pollinizer 2x (♂) (straight arrow), and a reciprocal cross (dash arrow) will also be performed. (**F**) The developed fruits will then be seedless.

The induction of parthenocarpy by exogenous plant growth regulators has already been reported for pointed gourd [2] and other cucurbits [3,4,5]. However, this practice is not genetically stable or economically feasible because it involves extra investment in labor and chemicals every year. Another approach to manage this problem is to develop triploid clones after crossing between tetraploid and diploid parents. From a horticultural point of view, such triploids are expected to be superior to their diploid counterparts, with a higher yield, more vigorous growth, and seedlessness or less-seeded characteristics, making them more attractive to consumers [6]. It has been reported that a tetraploid pumpkin fruit’s weight is 2.9 kg with fewer seeds (30), while that of diploids is 2.2 kg with more seeds (122) [7]. However, this technique is restricted for seed propagated species due to the inherent difficulties in maintaining genetic stability, as well as their low rate of viable seed production [8]. However, this seedlessness strategy does not affect the commercial utility and multiplication of pointed gourd because of vegetative propagation by vine cutting. It is hypothesized that colchicine treatment might be effective in inducing tetraploids in pointed gourd. These induced tetraploids (female or male) are assumed to be cross compatible with diploids (male or female), and their fruits would be less-seeded or seedless (Figure 2). In addition, if these fruits produce viable seeds, then these seeds will be F_1_ and could be used to determine the ploidy level. If these progenies are recognized as triploids, then it would be confirmed that colchicine-induced polyploidization in pointed gourd is genetically inheritable and that these triploids would be sterile. When the sterile female flowers cross with fertile diploid male flowers, the pointed gourd produced would be completely seedless. Once triploid line of the pointed gourd is developed, it can then be multiplied by vine cutting for commercial production and distributed to local nurseries. Further, if F_1_ seeds are not viable, then tetraploid lines will also be very important for producing less-seeded fruits while being cross-compatible with diploids. These facts will help facilitate the development of the tetraploid line as the logical first step for the expansion of genetic resources in further triploid breeding programs for pointed gourd. 

Colchicine is considered an effective chromosome doubling agent for inducing polyploidy in many vegetables [9]. Hazra [10] observed colchiploid induction in pointed gourd when 0.2% colchicine was applied to five-day-old seedlings, but this resulted in an abnormal morphology and an inability to produce flowers or fruit. However, the effectiveness of colchicine depends highly on the concentration applied, the duration of treatment, the type of explant, and the penetration of the compound [11]. 

These phenomena prompted us to hypothesize that colchicine, under various concentrations and soaking durations, might be effective for the seed treatment of pointed gourd for tetraploid induction. These tetraploids could be used as breeding parents for pointed gourd, similar to watermelon polyploidization, which is used to produce less-seeded or seedless fruit [12].

In this study, we examine the effectiveness of colchicine treatment under different concentrations and exposure times for tetraploid induction in pointed gourd seeds. We also follow full diallel approaches using diploid and induced tetraploid individuals (male or female) of *T. dioica* to determine their cross compatibility, focusing on fruit characteristics and seed production. This information will help plant breeders extend their genetic improvement studies using advanced genetic resources for dioecious pointed gourd.

## 2. Results

### 2.1. Effect of Colchicine Treatments on the Germination Rate and Surviving Seedling Rate 

In total, 42, 21, 25, and 45 seeds were germinated, and 35, 19, 19, and 41 seedlings survived in PGF02 × PGM03, PGF17 × PGM08, PGF18 × PGM08, and PGF19 × PGM09, respectively (Table 1). There was no consistent trend observed for concentrations or the duration of colchicine treatment on the germination and survival rate of pointed gourd seedlings. 

### 2.2. Confirmation of Ploidy Level

Colchicine treatments for 48 and 72 h at 0.5% concentration only resulted in 1 and 2 tetraploids in the PGF19 × PGM09 crossing seedlings (Table 2). No tetraploids were observed in any of the surviving seedlings of the other crosses. A total of 19 seedlings were confirmed to be mixoploids, but details on these plants are not shown in this study’s findings.

**Figure 3:**
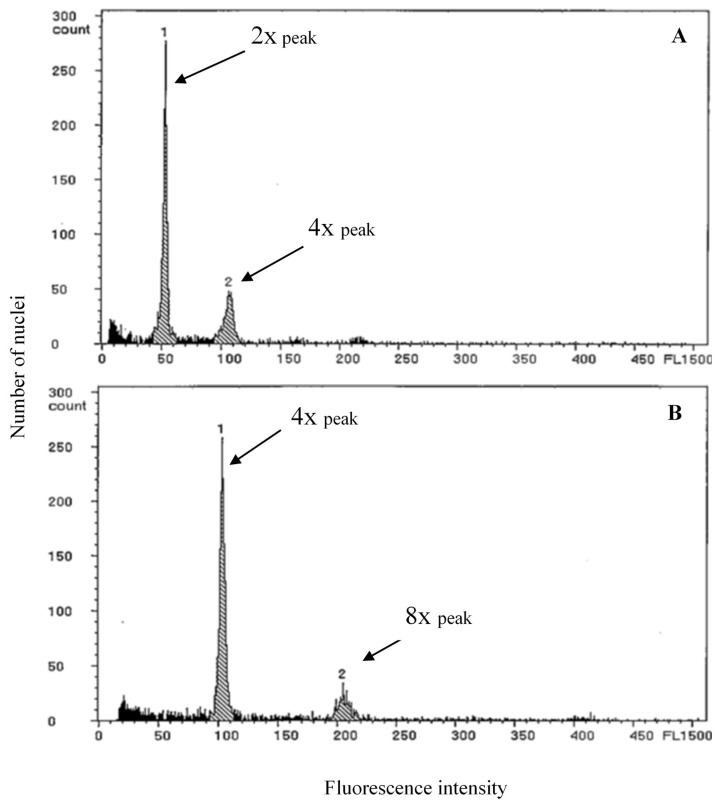
Histograms of the relative nuclear DNA content obtained from the flow cytometric analysis of the pointed gourd. (**A**) Diploid mother plant; (**B**) tetraploid of the PGF19 × PGM09 seedling.

The flow cytometric results presented in Figure 3 offer a representative fluorescence profile for the nuclei from normal diploid parent plants in comparison to colchicine treated tetraploid offspring. Two standard peaks of the diploid mother plants (female or male) appeared at about channel 50 and 100 with relative fluorescent intensity (Figure 3A). Hence, seedlings with two peaks at about channel 100 and 200 with relative fluorescent intensity were regarded as tetraploids (Figure 3B). 

### 2.3. Morphological Characteristics of Diploid and Colchicine-Induced Tetraploid Plants

Three tetraploid plants (confirmed after flow cytometry) were planted in the glasshouse at the same time as the diploids were planted. The morphological characteristics of the various vegetative and reproductive organs of the tetraploids were compared with those of their corresponding diploids (Table 3). The results revealed significant differences between the diploid and induced tetraploid lines for leaf length and diameter. Compared with the diploid parents, the lengths and diameters of the leaves were the highest among the tetraploids, with leaf lengths of 8.7 cm for tetraploids and 5.6 cm for diploids, and leaf diameters of 9.7 cm for tetraploids and 6.5 cm for diploids. However, the internode lengths and diameters did not differ significantly between the diploid and tetraploid individuals (Table 3). 

**Figure 4:**
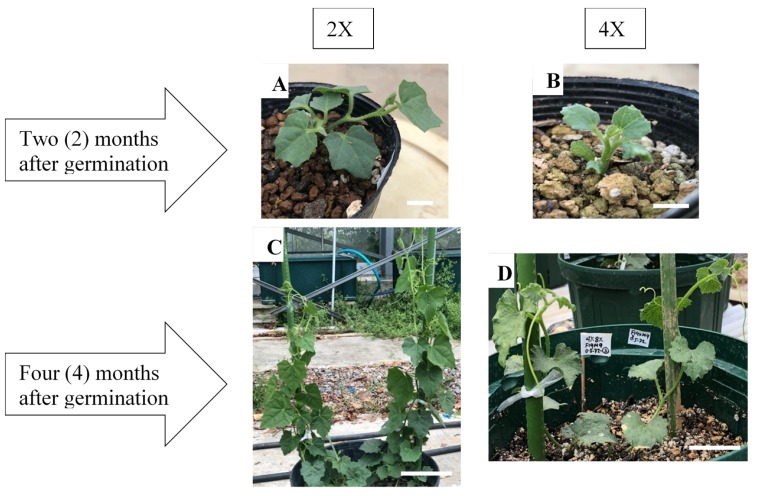
The morphological variation of the diploid and tetraploid pointed gourd seedlings that survived in 2018. (**A**), (**C**): diploid (2x), (**B**), (**D**): tetraploid (4x). Bars indicate 1 cm in A and B and 5 cm in C and D.

Considering their visual appearance, the tetraploid seedlings derived from a high concentration of colchicine coupled with a long exposure time possessed undesirable characteristics, such as stunted growth at two months after germination (Figure 4A,B). However, after four months, vigorous growth was observed in the tetraploids with rough leathery leaves and thick prominent vines, while smooth leaves and thin vines appeared in the diploid parents as the control (Figure 4C,D). 

**Figure 5:**
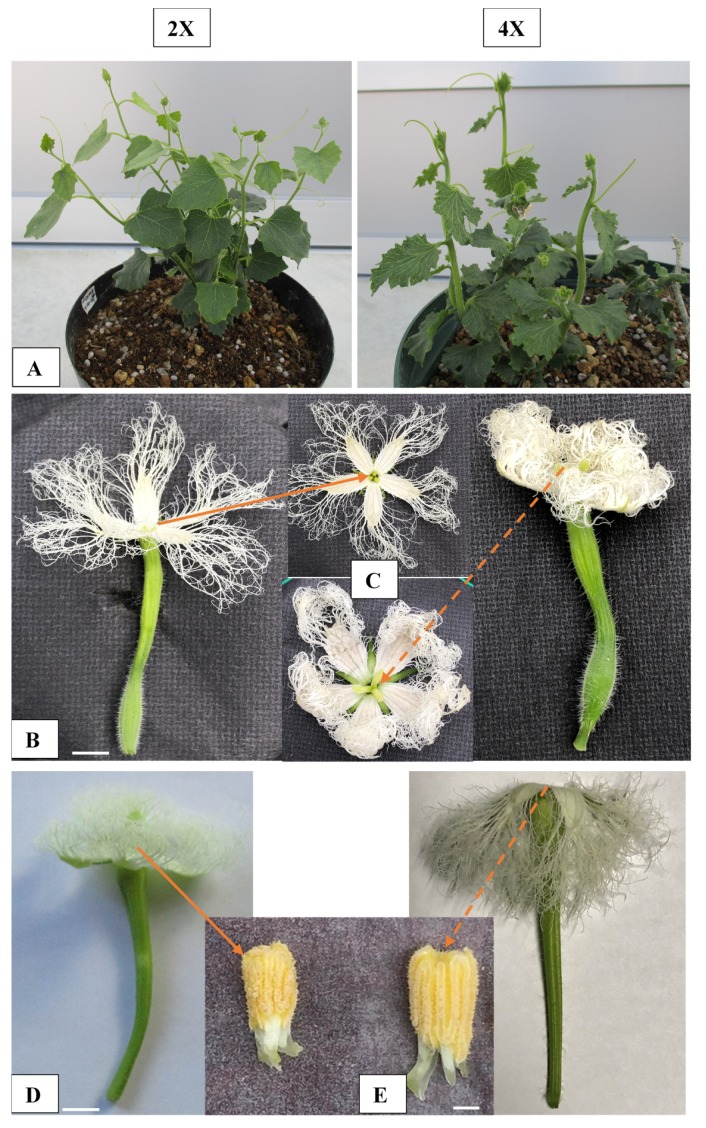
Vegetative and reproductive traits of the diploid and colchicine-induced tetraploid plants of pointed gourd grown in 2019. The diploid (2x) plant is presented on the left side and the tetraploid (4x) is on the right side. (**A**): Shoots regenerated from the previous year’s grown mature vine cuttings; (**B**): female flower; (**C**): petals and stigma; (**D**): male flower; (**E**): anther of the diploid plant (left side) showing smaller features compared to the colchicine induced tetraploid plant on the right hand side. Straight lines indicate the diploid stigma and anther; while the dashed lines indicate tetraploid stigmas and anthers. The bar indicates 1 cm (female flower), 5 mm (male flower), and 1 mm (anther).

Among the three induced tetraploids, two were differentiated as females and one as a male after flower bud initiation. The pistillate flower traits (Table 4) showed significant differences in both ploidy plants. Pistillate flowers of the tetraploid plant appeared at a comparatively higher node (30.3) than the diploids (12.7). The mean time to first flowering (74.3 days) for the diploid plant was significantly earlier than that of the tetraploid (129.7 days). For the tetraploids, their flowers were larger with bigger ovaries than their diploid counterparts. The ovary length and diameter were measured as 29.0 mm and 10.4 mm in tetraploids and 15.5 mm and 5.4 mm in the diploid pistillate flowers. The stigma structure proved the superiority of tetraploids (a prominent upward position) over diploids (a flat downward position) (Figure 5C). Both ploidy plants showed diploid pistillate flowers to have a lower petal length (19.5 mm) and diameter (6.3 mm) compared to the tetraploids (33.0 mm length and 17.6 mm diameter). However, the number of petals per flower (5) for the tetraploids was the same as that of the diploids (Figure 5B).

The staminate flower components in the diploid and tetraploid lines exhibited significant differences according to their ploidy levels. Stalk lengths among the diploid and tetraploid staminate flowers were 17.5 mm and 45.8 mm, respectively (Table 5). The tetraploid flowers showed a higher anther diameter (9.4 mm), but the number of anthers (3) was similar to that of the diploid genotypes. Petal length and diameter were the other prominent traits of the staminate flower and also demonstrate the increased size in the tetraploid compared to the diploid plants (Table 5). Staminate flowers (diploid and tetraploid) were used as the pollen donors for crosses with female flowers (diploid and tetraploid).

**Figure 6:**
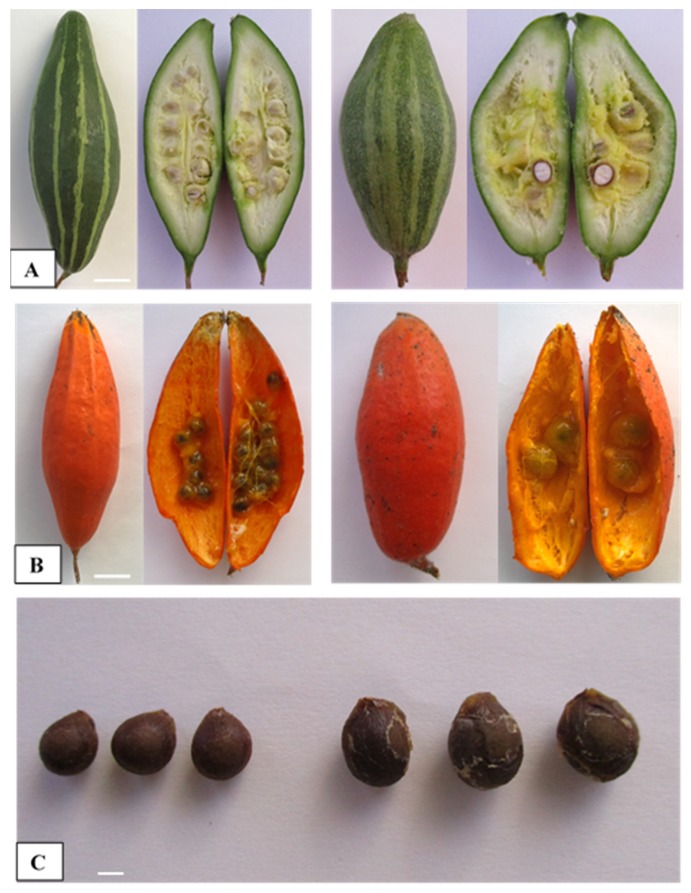
Fruit and seed traits after crossing between the diploid and colchicine-induced tetraploid of pointed gourd. A cross-section of the fruit at the green stage (**A**), the ripening stage (**B**), and mature seeds (**C**) produced from the 2x × 2x cross (left) vs. the 4x × 2x cross (right), which produce fruits with few but large seeds. Bar indicates 1 cm for fruit and 2 mm for seed diameter.

The fruit set rate was significantly different according to the ploidy levels of the parents who took part in the intraploidy and interploidy crosses (Table 6). No fruit development occurred when the diploid female flower was pollinated with a tetraploid male. When a tetraploid was used as a female parent in crosses with a tetraploid pollen donor, a small percentage (13.3%) of fruit was produced. However, these fruits experienced shrinkage, early yellowing, and failed to reach an edible stage. Meanwhile, in the tetraploid–diploid pair (4x × 2x), where the tetraploid female was involved, the maternal parent crossed with the diploid paternal parent produced fruits (93.3%) that were identical in their fruit set rate and shape compared to those of the original diploid (2x × 2x) (Table 6, Figure 6A), while the fruit length and weight were lower than those of the diploid. The fruit diameter (3.3 cm) was found to be almost same among all fruits produced from different cross combinations, with the exception of the 4x × 4x generated fruits (Table 6).

Surprisingly, remarkable variation was observed in the seed production, seed number, and size (diameter) between different crosses among the diploid and tetraploid lines, as presented in Table 7. None of the crosses where the diploid or tetraploid genotypes were used as maternal parents crossed with a tetraploid paternal parent were successful in producing viable seeds in our study. Further, the mature developed seeds in the fruits obtained from the crosses of 4x × 2x and 2x × 2x produced 1.8 and 26.4 seeds per fruit (Table 7, Figure 6B). Fruits of the 4x × 2x crosses yielded larger (7.8 mm diameter) seeds than the corresponding diploid 2x × 2x crosses (5.5 mm) (Figure 6C); the 100 seed weights for these aforementioned crosses were 12.4 and 6.4 g, respectively (Table 7). However, seeds of the 4x × 2x cross were unable to germinate, indicating their nonviability in nature, whereas 100% of seeds were germinated in the 2x × 2x cross.

## 3. Discussion

We reported the efficient in vivo induction of tetraploids in diploid *T. dioica* via the treatment of seeds with colchicine. Two months after germination, some seedlings died, experienced stunted growth, or failed to develop further true leaves with reduced survival rates in the treatments employing high colchicine concentrations, along with a long soaking period. This might have occurred due to a reduction in the seedling’s ability to overcome the inhibitory effect of colchicine on the living portion’s development at higher doses [13]. However, based on the flow cytometric analysis, we confirmed that healthy and actively growing tetraploids are produced from the highest concentration of colchicine at 0.5% for 48 h (one tetraploid) and 72 h (two tetraploids) seed imbibition in the PGF19 × PGM09 accessions (Table 1 and Table 2). From this finding, we determined that the colchicine concentration and duration are both important for tetraploid induction because colchicine is only effective in the cell dividing stage [14]. After colchicine treatment, colchicine likely penetrates into the seeds and remains inactive until the cells reach their active dividing stage. Meanwhile, for the equal polyploidization of all the vigorously grown treated cells, a greater concentration of colchicine is required. Therefore, a higher concentration and soaking duration was found to be effective in this study. 

The observation of seedlings after exposure to colchicine treatments revealed a number of morphological abnormalities. After germination, the first one or two true leaves on the tetraploid seedlings were brittle and sturdy, with stunted growth during the initial stage (Figure 4B). This might have resulted from the slowed mitotic divisions of larger cells with more chromosomes, thus prolonging the duration of their response to the growth stage [15]. However, four months after germination, the tetraploid plants recovered with vigorous growth and tended to have larger leaves compared to their diploid parents (Figure 4D). It is reported that colchicine may disrupt spindle fiber formation in mitosis after destructing the polymerization of microtubules. Afterwards, the cells affected at the metaphase of the cell cycle may recover and enter the next mitotic cycle with twice the number of chromosomes compared to the diploids [13]. Cells with a larger number of chromosomes grow larger to maintain a constant ratio of cytoplasm to nuclear volume and also express more proteins, which could be due to a higher expression of genes since their number of alleles is higher. This phenomenon accordingly contributes to an increase in plant organs. Colchicine-induced tetraploid watermelon plants, with broader leaves, bigger flowers, and few but larger seeds, served as the breeding parent in past triploid breeding programs [12,16], and our data recapitulate their findings. Although the internode length in the tetraploid was shorter than that in the diploid plants, the main vine was thicker in the tetraploid lines. The tetraploid plants induced flowering about two weeks later on the upper nodes than the diploids. This is due to the fact that the growth of the diploid plants until flowering was the most rapid, whereas the tetraploid plants were the slowest. Similar results, showing an increased size of the pistillate and staminate flower components associated with a higher ploidy level, were also reported in previous studies on various tetraploid plant species [12,17,18,19]. 

Fruit size is commercially important, as consumers pay a premium for larger fruit. Seedless or less-seeded fruit production while maintaining a standard fruit size for commercial marketing was a key goal of this study. The 4x × 2x cross produced fruits (8.8 cm length) were comparatively smaller than their diploid parents (11.0 cm) due to their remarkable reduction in the number of seeds per fruit. These fruits had only 1.8 observable seeds while their diploid parent had 26.4 seeds per fruit (Figure 6A). However, some misshapen fruits have been observed (featuring empty seeds with undeveloped endosperms) in other crosses where tetraploid parents were used as paternal pollen donors. This is possibly due to the pollen sterility in tetraploids and the paternal genomic imbalance, which particularly affects the proliferation of the endosperm [20,21]. The fruit size and seed formation differences between the 2x × 2x and 4x × 2x crosses are consistent with the previous observations of other plant species [12,22,23,24]. Previous studies noted that the cause of these differences is often irregularities in segregation or other abnormalities during meiosis, leading to reduced gamete production, pollen tube growth, or inhibition of fertilization. 

In the present study, we observed substantial variation in the seed sets in 4x × 2x cross fruits, showing only about 6.8% seeds compared to the 2x × 2x cross fruit (100%). This finding supports our hypothesis that the fruits derived from a cross of 4x × 2x have fewer seeds [25]. Informed tetraploid watermelons are partially sterile and generally produce only 25–40% as many seeds as the comparable diploid varieties. The low fertility among tetraploid plants is also likely related to the instability of the chromosome number during abnormal meiosis. Once established, the degree of fertility can be improved in successive generations [20]. Moreover, induced tetraploids are commonly used to produce seedless triploid hybrids for watermelon [12,26,27]. Therefore, the tetraploid females (seed parents) induced in this study are highly desirable in interploidy hybridization for the evolution of a new crop species with fewer seeds, which is highly beneficial to farmers and consumers. Although 4x × 2x did not produce viable seeds, the induced tetraploid female can easily be multiplied by vine cutting every year and used in further studies.

## 4. Materials and Methods

### 4.1. Colchicine Treatment 

Mature seeds of pointed gourd were collected in August 2017 from four different crosses (PGF02 × PGM03, PGF17 × PGM08, PGF18 × PGM08, and PGF19 × PGM09) and stored in a freezer at 5 °C before use in this experiment. The female parents (PGF02, PGF17, PGF18, and PGF19) and male parents (PGM03, PGM08, PGM09) were collected from Bangladesh and cultivated in Japan [28]. In October 2017, twenty-five (25) seeds from each cross were soaked in aqueous solution of 0.05%, 0.1%, 0.5% colchicine with 1% DMSO (Dimethyl sulfoxide) for 24, 48, and 72 h. The colchicine treated seeds were washed with distilled water three times and sown in vermiculite in a plastic tray. The trays were kept in a glasshouse at Hakozaki campus (lat. 33°37′ N; long. 130°25′ E), Kyushu University, Japan, until February 2018. Two months after germination (the 3–4 leaf stage), the seedlings were transplanted into plastic pots and cultivated in the same glasshouse. The average day and night temperature in the glasshouse ranged from 22 to 32 °C and 20 to 24 °C from February to October 2018. The percentage of germinated and surviving seedlings was assessed for each treatment.

### 4.2. Confirmation of Ploidy Level

Ploidy evaluation was conducted on the seedlings that survived six weeks after germination via flow cytometry analysis using a Partec Ploidy Analyzer flow cytometer (Sysmex-Partec, Germany). Young leaf tissue samples of approximately 1 cm^2^ from each treated seedling were chopped into small pieces with a razor blade in 400 μL nuclei extraction solution HR-A (CyStain UV Precise P, Sysmex-Partec High Resolution Staining Kit, Sysmex-Partec GmbH, Germany) and filtered through a 42 μm mesh filter (Sysmex-Partec CellTrics filter, GmbH, Germany) to remove debris. The suspension of the nuclei was stained with 1.6 ml of a HR-B DAPI staining solution (CyStain UV Precise P, Sysmex-Partec High Resolution Staining Buffer Kit, GmbH, Germany). A minimum of 7000 nuclei were measured per sample.

At first, 2–3 leaves from the control donor plants (male and female) without colchicine treatment were used to determine the standard peaks of the diploid mother cells of the pointed gourd. These standard peaks were used to compare the ploidy peaks produced by the colchicine treated seedlings. The identified tetraploid plants were then transplanted into 150 mm diameter plastic pots containing an akadama soil/peat mix (2:1) in the same glasshouse. At the same time, the diploid donor parents (male and female) were also planted into the same sized pots containing a soil mix identical to that described for the tetraploids to conduct a full diallel cross. 

### 4.3. Vegetative and Reproductive Traits

The morphological characterization of the diploid and tetraploid plants was carried out after 6 months at the fully vegetative growth stage during the first year of cultivation in 2018 and maintained until September. Then, the mature vines were used for multiplication. At this stage, the mean length and diameter of five randomly selected fully expanded leaves and internodes were recorded. These measurements were repeated three times. In February 2019, new shoots were generated from the previous year’s mature vine cuttings of the diploid and tetraploid plants (Figure 5A). These plants were transplanted from existing pots to new pots in the akadama soil/peat (2:1) mix and placed into glasshouse beds.

The compatibility between different crosses was evaluated in response to a phenotypic analysis of flowering, fruit characteristics, seed maturation, and germination. These characteristics were compared with those of the original diploid parents. Once the plants initiated flowering, individuals were monitored daily, and the time until the first flower was noted for each, along with the respective node number. The time to flowering was then estimated as the number of days between transplanting and the opening of the first flower on the plant. The pistillate and staminate flower components were measured using digital calipers during anthesis. The anthers in the staminate flowers were clustered so that their overall length and diameter could be measured. Similarly, the petal number, length, and diameter, ovary length and diameter, and visual appearance of the stigma structure of the pistillate flowers of the tetraploids were compared with those of the diploids. The fruit set rate (%), fruit length, diameter, weight, and number of developed and aborted seeds per fruit were recorded for 5 fruits of each cross. For the flower and fruit characteristic measurements, 5 flowers (female and male) and five fruits were evaluated for each replication; thus, a total of 15 flowers (female, male) and 15 fruits were used for the three replications. For seed characteristics, the total number of seeds (developed and aborted) in one fruit was counted in one replication, and a total of 3 fruits were evaluated for three replications in each cross. Finally, the average mean value was used for the analysis.

### 4.4. Crosspollination of Diploids with Colchicine-Induced Tetraploids

A total of 9 diploid plants from each female (PGF19) and male (PGM09) accession, 2 colchicine induced tetraploid female plants, and 1 tetraploid male plant were used as parents in the following crossing design. With the advent of flowering, each diploid was paired with a tetraploid and crossed reciprocally (i.e., all the diploids and tetraploids served as maternal and paternal parents). Five diploid and tetraploid female flowers were pollinated with pollen derived from the tetraploid and diploid male plants as an interploidy cross (2x × 4x, 4x × 2x). Each member of the pair was also pollinated on different days by the same ploidy as the intraploidy cross (2x × 2x, 4x × 4x). To ensure that there was sufficient pollen to fertilize all the available ovules, pollen from at least one flower with three anthers of the male donor was applied to the two female flowers, with each having three stigma lobes from the female parent.

Mature green fruits were harvested 15 days after pollination for a fruit characteristic analysis at the edible stage. However, some fruits were harvested at the ripening stage after 60 days of pollination for a seed characteristic analysis. To assess the effect of the cross treatments on seed maturation, the number of seeds (developed and aborted) was counted for each of the three fruits generated from each cross and compared with the total number of seeds produced in the normal cross. The total number of seeds in one fruit was counted for one replication, so a total of 3 fruits were evaluated for the three replications of each cross, and the mean data were used for the analysis.

### 4.5. Data Analysis

The experimental layout followed a randomized complete block design (RCBD), with three replications for most of the variables studied, while the colchicine-treated seed germination and polyploidy induction parameter data are represented as absolute values without following the replication process. The average mean data were evaluated by an analysis of variance, and the means were compared by honestly significant difference (HSD) test and t-test to determine the significant differences (*P* < 0.05) using R software [29].

## 5. Conclusions

We obtained pure tetraploid plants (female and male) by colchicine treatment (0.5% for 48 and 72 h) from pointed gourd seeds. These tetraploid plants displayed a number of significant differences in their vegetative and reproductive traits compared to the diploids due to their higher ploidy levels. Crossing between the tetraploid maternal parent and the diploid parental parent (4x × 2x) resulted in a successful fruit set with a remarkable reduction in the number of seeds per fruit, and seeds were larger than those of the diploid. To our knowledge, these results are the first preliminary evidence for the supporting role of colchicine induced tetraploids and well help pave the way toward further dynamic studies of triploid evolution in *T. dioica*. 

## Figures and Tables

**Table 1:** Effects of colchicine treatments on the germination rate and surviving seedling rate of pointed gourd.

Colchicine Treatments		PGF02 × PGM03		PGF17 × PGM08		PGF18 × PGM08		PGF19 × PGM09	
Conc. (%)	Duration (h)	Germinated Seeds (%)	Surviving Seedling (%) ^z^	Germinated Seeds (%)	Surviving Seedling (%)	Germinated Seeds (%)	Surviving Seedling (%)	Germinated Seeds (%)	Surviving Seedling (%)
0.05	24	1 (4.0)	1 (4.0)	3 (12.0)	2 (8.0)	2 (8.0)	2 (8.0)	1 (4.0)	1 (4.0)
	48	5 (20.0)	5 (20.0)	1 (4.0)	1 (4.0)	3 (12.0)	1 (4.0)	2 (8.0)	2 (8.0)
	72	12 (48.0)	9 (36.0)	4 (16.0)	4 (16.0)	0 (0.0)	0 (0.0)	9 (36.0)	9 (36.0)
0.1	24	1 (4.0)	1 (4.0)	2 (8.0)	1 (4.0)	5 (20.0)	5 (20.0)	7 (28.0)	7 (28.0)
	48	5 (20.0)	5 (20.0)	0 (0.0)	0 (0.0)	4 (16.0)	4 (16.0)	7 (28.0)	6 (24.0)
	72	8 (32.0)	6 (24.0)	3 (12.0)	3 (12.0)	2 (8.0)	2 (8.0)	5 (20.0)	4 (16.0)
0.5	24	1 (4.0)	1 (4.0)	5 (20.0)	5 (20.0)	2 (8.0)	2 (8.0)	3 (12.0)	2 (8.0)
	48	4 (16.0)	3 (12.0)	1 (4.0)	1 (4.0)	5 (20.0)	2 (8.0)	5 (20.0)	5 (20.0)
	72	5 (20.0)	4 (16.0)	2 (8.0)	2 (8.0)	2 (8.0)	1 (4.0)	6 (24.0)	5 (20.0)
Total		42	35	21	19	25	19	45	41

25 seeds were used in each treatment. ^z^ Surviving seedlings were counted two (2) months after germination.

**Table 2:** Effects of colchicine treatments on the induction of polyploid seedlings in pointed gourd.

Colchicine Treatments		PGF02 × PGM03			PGF17 × PGM08			PGF18 × PGM08			PGF19 × PGM09		
Conc. (%)	Duration (h)	Ploidy Levels ^z^			Ploidy Levels			Ploidy Levels			Ploidy Levels		
		2x	4x	2x + 4x	2x	4x	2x + 4x	2x	4x	2x + 4x	2x	4x	2x + 4x
0.05	24	1	0	0	2	0	0	2	0	0	1	0	0
	48	4	0	1	1	0	0	1	0	0	2	0	0
	72	8	0	1	4	0	0	0	0	0	9	0	0
0.1	24	1	0	0	1	0	0	5	0	0	7	0	0
	48	4	0	1	0	0	0	3	0	1	6	0	0
	72	3	0	3	2	0	1	1	0	1	4	0	0
0.5	24	1	0	0	5	0	0	2	0	0	2	0	0
	48	3	0	0	0	0	1	0	0	2	3	1	1
	72	3	0	1	1	0	1	0	0	1	0	2	3
Total		28	0	7	16	0	3	14	0	5	34	3	4

^z^ Ploidy levels denoted as 2x = diploid, 4x = tetraploid, [2x + 4x] = Mixoploid.

**Table 3:** Comparison of the morphological characteristics of colchicine-treated pointed gourd seedlings with different ploidy levels.

Ploidy Level	No. of Evaluated Plants	Leaf Length (cm)	Leaf Diameter (cm)	Internode Length (cm)	Internode Diameter (mm)
Diploid (2x)	3	5.6 ± 0.2 b ^z^	6.5 ± 0.1 b	9.9 ± 1.0 ^ns^	2.6 ± 0.1 ^ns^
Tetraploid (4x)	3	8.7 ± 0.1 a	9.7 ± 0.1 a	8.6 ± 0.5	3.6 ± 0.5

Five randomly selected leaves and internodes were measured from each shoot, and three shoots were considered for three replications; ns = Not significant. ^z^ Values are the means ± standard error; the column means under each parameter with the same letter (s) are not significantly different at *P* < 0.05, as determined by a t-test using the R software.

**Table 4:** Comparison of the pistillate flower traits in induced tetraploids with diploid pointed gourd plants.

Ploidy Levels ^z^	No. of Days to First Flowering	Node No. of First Flowering	Flowers No./ Plant ^y^	Ovary Length (mm)	Ovary Diameter (mm)	Stalk Length (mm)	Petal Length (mm)	Petal Diameter (mm)
2x	74.3 ± 1.5 b ^x^	12.7 ± 1.1 b	51.0 ± 2.0 a	15.5 ± 1.1 b	5.4 ± 0.6 b	28.5 ± 0.9 b	19.5 ± 0.8 b	6.3 ± 0.6 b
4x	129.7 ± 3.2 a	30.3 ± 2.3 a	31.7 ± 2.5 b	29.0 ± 1.2 a	10.4 ± 1.0 a	31.1 ± 0.8 a	33.0 ± 1.2 a	17.6 ± 1.0 a

^z^ Ploidy levels denoted as 2x = diploid, 4x = tetraploid. ^y^ Five pistillate (female) flowers were evaluated for each replication; a total of 15 flowers were used for three replications in three plants for each diploid and tetraploid plant. ^x^ Values are the means ± standard error; the column means under each parameter with the same letter (s) are not significantly different at *P* < 0.05, as determined by a t-test using the R software.

**Table 5:** Comparison of the staminate flower traits in the induced tetraploid with diploid pointed gourd plants.

Ploidy Levels ^z^	Stalk Length (mm)	Stalk Diameter (mm)	Anther Length (mm)	Anther Diameter (mm)	No. of Anthers	Petal Length (mm)	Petal Diameter (mm)
2x	17.5 ± 0.9 b ^y^	3.6 ± 0.6 b	3.1 ± 0.1 b	5.1 ± 0.2 b	3.0 ± 0.0 ns	16.1 ± 0.6 b	5.6 ± 0.1 b
4x	45.8 ± 1.2 a	6.0 ± 0.9 a	4.5 ± 0.2 a	9.4 ± 0.3 a	3.0 ± 0.0	24.5 ± 0.5 a	14.1 ± 0.3 a

^z^ Ploidy levels are denoted as 2x = diploid and 4x = tetraploid. Five staminate (male) flowers were evaluated for each replication; a total of 15 flowers were used for three replications in three plants of each diploid and tetraploid plant. ^y^ Values are the means ± standard error; the column means under each parameter with the same letter (s) are not significantly different at *P* < 0.05, as determined by a t-test using the R software.

**Table 6:** Comparison of the fruit traits in different cross combinations between the diploid and colchicine-induced tetraploid pointed gourd lines.

Cross Combination ^w^ (Seed Parent × Pollen Parent)	No. of Flowers Pollinated/Repetition ^z^	No. of Flowers that Set Fruits	Fruit Set Rate (%)	Fruit Length (cm)	Fruit Diameter (cm)	Fruit Weight (g)
2x × 2x	5	5.0 ± 0.0 a ^y^	100.0 ± 00.0 a	11.0 ± 0.9 a	3.3 ± 0.0 a	45.7 ± 2.3 a
2x × 4x	5	– ^x^	–	–	–	–
4x × 4x	5	0.7 ± 0.6 b	13.3 ± 11.5 b	2.9 ± 0.1 c	0.9 ± 0.8 b	4.3 ± 3.7 c
4x × 2x	5	4.7 ± 0.5 a	93.3 ± 11.5 a	8.8 ± 0.5 b	3.3 ± 0.2 a	33.4 ± 0.9 b

^z^ Five (5) female flowers were pollinated per cross in one replication; a total of 15 female flowers were pollinated for three replications. ^y^ The values are the means ± standard error; the column means under each parameter with the same letter (s) are not significantly different at *P* < 0.05, as determined by Honestly significant difference test using the R software. ^x^ Dashes indicate that no fruit setting occurred. ^w^ Ploidy levels are denoted as 2x = diploid and 4x = tetraploid.

**Table 7:** Comparison of the seed traits in different cross combinations between the diploid and colchicine induced tetraploid pointed gourd lines.

Cross Combination ^z^ (Seed Parent × Pollen Parent)	No. of Evaluated Fruits ^y^	No. of Developed Seeds	No. of Aborted Seeds	Seed Diameter (mm)	100 Seeds Weight (g)	Seed Germination (%)
2x × 2x	3	26.4 ± 0.9 a ^x^	1.3 ± 0.5 b	5.5 ± 0.1 b	6.4 ± 0.3 b	100.0
2x × 4x	3	– ^w^	–	–	–	–
4x × 4x	3	0.0 ± 0.0 b	1.0 ± 0.9 b	–	–	–
4x × 2x	3	1.8 ± 0.0 b	2.7 ± 0.3 a	7.8 ± 0.2 a	12.4 ± 0.6 a	–

^z^ Ploidy level is denoted as 2x = diploid and 4x = tetraploid. ^y^ The total number of seeds in one fruit was counted in one replication; a total of three fruits were evaluated for three replications in each cross. ^x^ The values are the means ± standard error; the column means under each parameter with the same letter (s) are not significantly different at *P* < 0.05, as determined by Honestly significant difference test using the R software. ^w^ Dashes indicate that no seed production or germination was observed.
